# Adverse Drug Interaction Between Kratom and Amitriptyline With Gastrointestinal and Mild Hepatic Effects

**DOI:** 10.7759/cureus.33809

**Published:** 2023-01-15

**Authors:** Naisarg B Vanani, Stephen G Stevanovic, Nebojsa Stevanovic

**Affiliations:** 1 College of Medicine, Medical College of Wisconsin, Milwaukee, USA; 2 Family Medicine, Ascension St. Francis Hospital, Milwaukee, USA

**Keywords:** adverse side effect, pharmacology, kratom, elevated liver enzyme, medication interaction, stress and depression, chronic and acute pain management, kratom addiction, substance abuse program, opioid use disorder (oud)

## Abstract

Kratom (*Mitragyna speciosa*) is a tropical evergreen plant native to Southeast Asia, where it has been used historically for its various psychoactive and analgesic properties. In recent years, the popularity of kratom has surged in the United States as a supplement for treating opioid withdrawal/addiction, anxiety, depression, and chronic pain, among others. However, much of Kratom processing and sales remain largely unregulated, with little clinical research to demonstrate the effects of kratom on physiologic processes such as potential drug interactions. Here, we present a case of Kratom interaction with Amitriptyline in a patient recovering from Opioid Use Disorder.

## Introduction

Kratom (*Mitragyna speciosa*) originates from Southeast Asia, where it has been used for centuries for its psychotropic, stimulant, and analgesic properties for the treatment of various ailments, such as chronic pain, opium addiction, and gastrointestinal upset [1,2]. In countries such as Malaysia, Myanmar, and the Philippines, Kratom is cultivated in leaf form and is often consumed in tea or chewed for its uplifting effects to treat fatigue or even sexual enhancement [2,3]. 

The popularity of Kratom in the United States has drastically increased in the last decade, which is evident in the 10-fold increase in Kratom-related poison center calls from 2010 to 2015 [4]. A 2019 study of a nationally representative sample demonstrated that approximately 0.7% of the US adult population, or 2 million people, had used Kratom that year [5]. Much of its popularity in the US can be attributed to its promulgation as a cure-all solution to opioid addiction, chronic pain, anxiety, and more, despite lacking any formal United States Food and Drug Administration (FDA) approval or institutional backing [6]. Additionally, except in the six states where it remains banned, Kratom is readily available in various forms, such as a whole leaf, concentrated extract, or capsular form, at gas stations, supplement stores, smoke shops, and online stores [6,7]. 

Kratom's pharmacological profile contains a myriad of phytochemical compounds, specifically 40 previously identified alkaloids, of which 7-hydroxymitragynine and mitragynine (MTG) produce most of the plant’s psychotropic effects [2]. The effects of Kratom and its alkaloid compounds are variable, with lower doses of up to 5 grams producing stimulant-like effects and high doses of greater than 15 grams producing effects similar to opioids' sedative properties [8]. However, reports have associated Kratom with severe addiction, along with adverse drug reactions such as exacerbated psychiatric conditions, cardiac arrhythmia, respiratory depression, withdrawal symptoms, liver injury, and death [8,9].

Previous literature demonstrates the predominantly liver metabolism of Kratom’s alkaloid compounds processed by cytochrome p450 (CYP) enzymes. Hepatic metabolism of these compounds lead to the inhibition of CYP enzymes, particularly CYP2D6, which is responsible for the metabolism of ~20% of clinically used drugs [10]. Amitriptyline is a tricyclic antidepressant medication that is processed by CYP2D6, presenting the possibility of drug-to-drug interaction (DDI) between CYP2D6 inhibitor and amitriptyline [11]. Here, we present a case of a 37-year-old man with a history of Opioid Use Disorder (OUD) and depression that shows evidence of the possibility of drug-to-drug interaction between Kratom and Amitriptyline.

## Case presentation

A 37-year-old Caucasian male with a history of major depressive disorder (MDD) and OUD presents to the clinic with complaints of three weeks of fatigue, dry mouth, dry eyes, lower back pain, constipation, generalized abdominal discomfort, and occasional nausea that has been progressively worsening. He described his xerostomia as a sticky sensation and stated that he had been having 1-2 bowel movements per week minimally, relieved by Miralax (polyethylene glycol 3350), when he typically has at least one movement per day. He did not report any syncope, fever, chills, diarrhea, headache, suicidal ideation, homicidal ideation, or any other symptoms. The patient had been previously treated at our clinic, where he was on Suboxone therapy for 18 months and had completed Suboxone (Buprenorphine/naloxone) treatment four months before this presentation. Shortly after this, he reports that he was helping his brother move out of state and was helping with home improvements on his new home. The patient initially denied any drug use, alcohol use, or lifestyle changes during the history. He also stated that he has been adherent with his current medications and supplements, including amitriptyline 75mg tablet at bedtime, which he has been using for the past 15 years for his depression. 

When asked for a more detailed history of the patient’s daily activities while at his brother’s home, he admitted to beginning the use of Kratom. He states that while working at his brother's home, he strained his back and began to have severe back pain and that his brother, an avid supporter of herbal therapy, advised him to try Kratom in oral and capsular form. The patient describes that he would initially use Kratom on an as-needed basis, using it when he needed it to get through the work pain-free. However, what started as a 3 g dose gradually increased in both dosage and frequency as the patient stated he needed more for the same effect. During his last week at his brother’s home, he was routinely taking Kratom for sleep and estimated taking an excess of 12-14 g/day in capsular form. It was during this last week when his initial symptoms of xerostomia, dry eyes, and constipation first began, which has progressively worsened in the past two weeks that he has returned home.

Labs taken five days before the visit showed no evidence of anemia or vitamin deficiencies (Vitamin D, B12, Folate) that could be contributing to his fatigue. Vital signs were normal (Blood Pressure: 115/70 mmHg, Pulse: 73 bpm, Temperature: 97.9°F, Respiration Rate: 14/minute). However, his comprehensive metabolic panel (CMP) was indicative of mildly elevated bilirubin and liver enzymes, with alanine transaminase (ALT) of 67 U/L (previously 23 U/L six months prior), aspartate transaminase (AST) of 31 U/L, alkaline phosphatase ALP of 110 U/L, and total bilirubin of 1.3 mg/dL (previous value 0.7 mg/dL). On physical exam, the patient appeared generally cooperative and well, awake, alert, and oriented to time, place, person, and event. There were no obvious signs of withdrawal or overdoses, such as tremors or stupor. Bowel sounds were hypoactive, with a palpable descending colon consistent with constipation. There was no abdominal tenderness to palpation, and signs of salivary gland obstruction or swelling were absent. As the only recent change since his last visit has been Kratom use and the absence of evidence pointing to other etiologies, the patient was counseled on ceasing the use of Kratom. The patient was informed that compounds from Kratom are processed in the liver, and potential drug interaction of said compounds with amitriptyline could be precipitating acute liver injury and his symptoms of xerostomia, constipation, and nausea, which are documented side effects of amitriptyline. The patient was sent home with instructions to continue amitriptyline as before and cease Kratom use. He was advised to return in 2-3 weeks with repeat labs, with instructions to present to the emergency room with any emergent concerns. 

On the follow-up appointment three weeks later, liver enzyme levels had normalized, and the patient reports that he is back to 1-2 bowel movements per day with regular stool consistency and has regained normal salivation in the past two weeks. However, he stated that when he ceased Kratom use, he began to have severe withdrawal symptoms within the first week. He described his symptoms to be similar to those he felt when he was in withdrawal from opioid use, including anxiety, generalized aches and pains, insomnia, and worsening cravings. It was at this point that the most probable diagnosis was made of adverse drug reaction of amitriptyline secondary to DDI between Kratom and amitriptyline. Options for withdrawal management were discussed, including counseling, which had been previously successful for the patient.

## Discussion

This is a case of a 37-year-old male with a history of MDD and OUD presenting with effects of potential DDI between Kratom and amitriptyline after taking progressively increasing intake of Kratom over 12 weeks. Considering the patient's consistent use of amitriptyline for over a decade without adverse side effects along with the timeline of symptom onset, a strong case can be made for a DDI brought on by Kratom, resulting in the anticholinergic effects of amitriptyline to precipitate. It is important to mention that Kratom itself has been shown to cause effects such as intermittent nausea, stupor, agitation, sedation, delirium, pruritis, constipation at low/short-term doses with tremors, psychosis, hyperpigmentation, hair loss, liver toxicity, and insomnia seen with long-term high dose use [12]. In our case, the patient began to experience the symptoms of xerostomia, constipation, and eye dryness with sustained and long-term high-dose causing these persistent effects. While intermittent xerostomia and constipation have been attributed to short-term low-dose Kratom use, the patient, in this case, had been using Kratom for nine weeks with increasing dosage before developing these progressively worsening symptoms. 

Since the patient developed characteristically anticholinergic symptoms of xerostomia, constipation, and dry eyes in the absence of common signs of Kratom toxicity such as aggression, delirium, tremors, stupor, and seizures, the possibility of a compounding DDI between Kratom and amitriptyline was considered [12]. Common adverse drug reactions of amitriptyline include xerostomia, dry eyes, urinary retention, constipation, and cognitive impairments [13]. These are a concern for those who are genetically CYP2D6-poor metabolizers; the CYP450 enzyme isoform is heavily involved in amitriptyline metabolism [11]. As the patient has been taking a consistent dose of amitriptyline for 15 years, it is unlikely that the results of this sudden poor metabolism of CYP2D6 are due to a genetic etiology. Kratom’s alkaloid compounds, such as MTG and corynanthidine (COR), have been shown to potently inhibit CYP2D6, providing a likely etiology of CYP2D6 poor metabolism phenotype that could have led to the anticholinergic side effects of amitriptyline seen in this patient [10]. Thus, physicians need to be aware of the onset of anti-cholinergic symptoms and their correlation with the patient’s more regular and higher dose intake of Kratom. 

As the various alkaloid compounds found in Kratom are heavily metabolized by the liver, heavy Kratom use also can result in drug-induced liver injury (DILI) in rare instances, a complication that has been reported in a previous case series where one patient presented with ALT of 404 U/L and AST of 345 U/L [9]. Thus, while the patient’s ALT and total bilirubin in our case were mildly elevated compared to those seen in this previous case, it is possible that our patient was at the beginning stages of a progressively worsening DILI. What is more concerning is that as an unregulated supplement, the alkaloid profile of Kratom can vary from batch to batch and between different strains, making potential adverse side and psychotropic effects of the drug further unpredictable [2]. This presents a potential challenge to physicians in the diagnosis and workup of Kratom-induced pathologies and warrants attention to detailed history taking, as patients may minimize Kratom as a mere herb whose use doesn’t merit disclosure to their physicians. In this case, a more detailed history revealed the patient's use of Kratom, which was not disclosed with the standard question of inquiring about new drugs or substance use. 

It is important to discuss the unique pharmacologic properties of Kratom's action effects on pain physiology. Previous literature indicates that the MTG alkaloid found in Kratom has both anti-inflammatory and antinociceptive properties [12]. More specifically, MTG has been shown to suppress cyclooxygenase-2 expression, leading to reduced prostaglandin E2 levels and contributing to Kratom’s anti-inflammatory effects [14]. Additionally, a study using a murine model demonstrated MTG’s effects on the stimulation of 5-hydroxytryptamine and noradrenaline release in descending pathways of the spinal cord, demonstrating MTG’s potential contribution an anti-nociception in humans [12,15]. Previous studies have demonstrated that MTG and 7-OH-MTG have a considerable affinity for the opioid receptor, with 7-OH-MTG’s affinity being more than 10-fold that of morphine [1]. A model for MTG and 7-OH-MTG’s action at the opioid receptors suggests that MGT is selective on µ- and δ-receptors and 7-OH-MTG have more effects on µ- and κ-receptors [16]. For this reason, while Kratom displays antinociceptive effects like opioids, it also has high addiction potential. 

With increasing rates of Kratom use in the US in the past decade, further research is needed on treatment and management options for Kratom dependence and withdrawal. Based on these mechanisms of action of Kratom alkaloids, previous literature suggests that Suboxone is a viable treatment for Kratom replacement therapy, which was considered for the patient in this case [17]. Additionally, based on the pharmacology of Kratom, there are concerns for opioid resistance, creating challenges for administering anesthesia during procedures. A recent case report discusses a heavy Kratom user who demonstrated profound resistance to standard anesthetics and suffered from poor pain control post-operatively to the point of delirium [18]. Thus, much research is needed to develop guidelines for addressing Kratom use, dependence, and the complexity of pain management in patients with Kratom-induced opioid tolerance.

## Conclusions

As increasing amounts of patients seek alternative treatments for opioid addiction, Kratom is often touted as a seemingly harmless herbal remedy that has become as easy to acquire as a simple click on a computer screen. As this case demonstrates, the pharmacology of Kratom is quite complex and variable, compounded by a largely unregulated market that poses a legitimate danger for those who choose to self-medicate without proper physician guidance. This warrants the need for further research on this topic and demonstrates the value of taking a meticulous history when it comes to the use of supplements. Physicians must be aware of the potential for DDIs with Kratom and the various implications of Kratom use in patients with regard to the prescription of new medications, perioperative pain management, and treatment of opioid use disorder. 
